# Weight-Related Behavior among Adolescents: The Role of Peer Effects

**DOI:** 10.1371/journal.pone.0021179

**Published:** 2011-06-23

**Authors:** Mir M. Ali, Aliaksandr Amialchuk, Frank W. Heiland

**Affiliations:** 1 Department of Economics, University of Toledo, Toledo, Ohio, United States of America; 2 Office of Regulations, Policy and Social Science, Food and Drug Administration, College Park, Maryland, United States of America; 3 Department of Economics, University of Toledo, Toledo, Ohio, United States of America; 4 City University of New York Institute for Demographic Research, School of Public Affairs, City University of New York Graduate Center (Economics), Baruch College, New York City, New York, United States of America; University of Zaragoza, Spain

## Abstract

**Purpose:**

To investigate whether social interactions in friendship networks influence the following weight-related behaviors of adolescents: exercising regularly, playing an active sport, hours of TV/Video viewing, sleeping six or fewer hours, eating breakfast on weekdays, frequency of eating at fast food restaurants, eating five servings of fruits/vegetables daily, and consuming calorie-dense snacks.

**Method:**

Data from a nationally representative sample of adolescents are used to examine the association between peer and individual weight-related behaviors. Evidence from multivariate regression analysis controlling for an extensive list of individual- and family-level factors as well as school-level unobserved heterogeneity is obtained.

**Results:**

We find a significant positive association between individuals' and friends' behaviors in terms of sports, exercise and fast food consumption. The estimated associations are robust to controls for individual- and family-level factors, unobserved heterogeneity at the school level and our attempts to account for non-random peer selection.

**Conclusions:**

The social transmission of weight-related behaviors is a viable explanation for the spread of obesity in friendship networks documented in recent research. Traditional weight reduction interventions may be fruitfully complemented with strategies that focus on harnessing peer support to modify behaviors.

## Introduction

Excess body weight among children and adolescents is one of the most pressing health problems today. The percentage of overweight children age 6–11 has more than doubled and the percentage of overweight adolescents age 12–19 has more than tripled since late 1970s [1]. In 2003–2004, 37.2% of children ages 6 to 11, and 34.3% of adolescents ages 12 to 19 were overweight or at risk for overweight [2].

Since the dramatic increase in average weight and obesity has occurred in genetically stable populations, the weight gains can be attributed to environmental factors related to calorie intake and/or physical activity. Indeed, poor dietary choices and health behaviors such as skipping breakfast, a diet low in fruits and vegetables, eating at fast food restaurants and consuming calorie-dense snacks, all of which are associated with a risk of abnormal weight-gain and adiposity among children and adolescents, have been on the rise [3]–[6].

Children's consumption of calories from fast food is estimated to have risen from 2% of total energy intake in the late 1970s to 10% of energy intake in the 1990s [7]. Between 1965 and 1991, breakfast consumption by children ages 8 to 10 and adolescents declined by 9% and 13%, respectively [8]. Furthermore, while the average size of a snack and the caloric energy per snack have remained relatively constant between 1977 and 1999, the frequency of snacking among children increased significantly [9].

In light of evidence that sedentary behaviors such as watching TV/video are associated with obesity [10]–[13], researchers have argued that the lack of opportunity to engage in physical activity in schools and the growing availability of more sedentary alternatives as opposed to traditional sports-related leisure pursuits may have contributed to the rising prevalence of overweight among children and adolescents. While surveys of adolescents, beginning in the 1990s, have suggested little or no decrease in the level of physical exercise across those cohorts and a relatively stable level of sedentary activities [14]–[16], the long-term trends in how adolescents use time and their level of physical activity is unclear because of the lack of comparable data.

Social interactions may also have contributed to the rapid rise in obesity [17], [18]. Although it is difficult to talk about causality, a number of observational studies have used data on the social networks of adults to document a positive correlation between adults' weight and the average weight of their peers [18]. Several studies reached similar results in terms of adolescents' weight and the weight of their peers [19]–[22]. However, the nature of the social interactions that cause the apparent spread of obesity in social networks is not well understood. Burke and Heiland [17] considered the possibility of interactions on weight through social weight norms and showed how an increase in the reference group's average weight can result in additional calorie consumption and weight gain. Individuals' lifestyle choices may also be directly influenced by peer behavior. For example, as regular exercise and sports become more popular in an individual's social network, the chances that the individual also engages in these pastimes increases, perhaps because of interest stimulated by the peer involvement in this activity or social pressures to conform to the group behavior.

In addition to the evidence supporting social interaction on weight, peer influences have been found in various health-related behaviors, ranging from smoking [23], [24], alcohol use [25], other health risky behaviors [26], [27], to health care utilization and preventative care [28]. To date, much of the attention to the social transmission of weight-related behaviors among children and adolescents has been on eating disorders [29]–[31]. A notable exception is the study by Fletcher [32], who analyzed peer effects on adolescents' time spent watching TV.

However, estimates of the relationship between social networks and health behaviors have been viewed with skepticism, largely from the failure to address the issues of peer selection and environmental confounders [19], [20], [27]. Peer selection implies that the correlation in behavior could be attributed to the similarity among individuals, whereas peer influence implies that the correlation is due to the peer behavior itself. Disentangling the peer influence from spurious unobserved factors associated with peer selection is important if we are to accurately estimate the relationship between the behaviors of the individual and that of their peers. Thus, if there are common underlying attributes of individuals within a peer group that drive behavior more than peer influence itself, policies aimed at taking advantage of peer influence may not realize the desired effects [23], [25], [26]. However, it is important to note that there is disagreement in the literature as to how to adjust for peer selection and choice of the methodology to account for this in most cases is driven by the data set that is being utilized in the study [27]. We elaborate further on this point in the Statistical Analyses section when we discuss our attempt to account for peer selection.

Environmental confounders, on the other hand, refer to factors that might be common to all adolescents living in the same environment, or community-specific factors influencing the outcome of all individuals in the same reference group. These effects, when unmeasured, can lead us to incorrectly attribute social network effects in individual outcomes when none might exist. For example, a higher concentration of fast-food restaurants or scare public recreational facilities in a neighborhood could simultaneously affect the eating pattern or exercise habits of all individuals in networks within the community. Thus, caution should be exercised in attributing causality to correlations in outcomes between friends when environmental confounders are not adequately accounted for.

The present research provides a comprehensive investigation of the relationship between peer and individual weight-related behaviors among adolescents. We consider seven previously unexamined behaviors related to physical activity and diet (energy expenditure and intake): regular physical exercise, participation in a sport, eating breakfast, eating at fast food restaurants, consuming five servings of fruits/vegetable daily, calorie-dense snacking, and six or fewer hours of nightly sleep. In addition, we analyze whether there are interaction effects in TV viewing in networks of close friends, which complements Fletcher's [32] study of TV viewing behavior among schoolmates. The current study builds on previous research supporting peer effects related to obesity in general by looking at specific behaviors that may be responsible for the spread of obesity in social networks. We hypothesize that behaviors that are easily observable by other adolescents are better candidates for peer interaction effects. Hence, individual sleep habits and breakfast consumption, which are not directly observable by peers, should be less likely to be influenced by the respective peer behaviors than participation in sports, exercise, or eating at fast food restaurants, which are activities that directly benefit from peer participation.

## Materials and Methods

### Data Source

The data for this study are drawn from the National Longitudinal Survey of Adolescent Health (henceforth “Add Health”). Add Health surveyed adolescents in grades 7 to 12 in 132 schools nationwide in the U.S. Beginning with an in-school questionnaire administered to a nationally representative sample of students in grades 7 through 12 in 1994–95 (Wave 1), the study follows up with a series of in-home interviews of respondents approximately one year (Wave 2; 1996), six years (Wave 3; 2001–2002), and thirteen years later (Wave 4; 2007–2008).

A unique feature of Add Health is that the first two waves (1994–95 and 1996) contain information on individuals' nominations of their closest friends. Since these friends were also surveyed, peer measures of weight-related behaviors can be constructed from actual responses. We also employ parental information from the parent questionnaire administered in the in-home survey in the first wave. (A full description of the sample design, data and documentation is available at www.cpc.unc.edu/addhealth.)

The samples in this study are drawn primarily from the wave II (1996) respondents in grades 7 through 12 with at least one nominated friend. Where possible, we link data from the first and second wave of the in-home survey (not all respondents were interviewed in both waves, and some nominated friends were not surveyed).Our analysis is based on samples of 3,898 adolescents and their peers. The average number of nominated friends per individual is 2.54, and approximately 85% of the friends are from the same school as the respondent. Detailed summary statistics on adolescent and peer weight-related behaviors for our analysis are in Table 1, while Table 2 reports the corresponding descriptive statistics for the control variables. The measures that we constructed from wave I (1994–95) data and linked to the adolescents interviewed in 1996 are noted in the tables.

**Table 1:** Descriptive Statistics (Adolescents from Add Health Wave II/1996).

Variables	Mean	Std. Dev.	Min	Max	N
*Weight–Related Behaviors*					
BMI (1994)	22.473	4.509	12.016	56.384	3898
BMI	23.157	5.135	14.042	51.686	3898
Exercise	0.516	0.500	0	1	3898
Sports	0.445	0.497	0	1	3898
Hrs of TV	14.597	14.181	0	162	3898
Sleep six or fewer hours	0.132	0.338	0	1	3898
Breakfast	0.863	0.344	0	1	3898
Fast Food	2.172	1.727	0	7	3898
Five Servings of Fruits or Vegetables	0.337	0.473	0	1	3898
Calorie-Dense Snack	0.518	0.500	0	1	3898
*Peer Variables*					
BMI (1994)	22.178	3.312	13.312	42.068	2760
BMI	22.731	4.061	12.692	51.686	3898
Exercise	0.518	0.432	0	1	3898
Sports	0.460	0.438	0	1	3898
Hrs of TV	13.970	11.800	0	162	3898
Sleep six or fewer hours	0.058	0.209	0	1	3898
Breakfast	0.868	0.292	0	1	3898
Fast Food	2.261	1.509	0	7	3898
Five Servings of Fruits or Vegetables	0.328	0.407	0	1	3898
Calorie-Dense Snacks	0.510	0.432	0	1	3898

**Table 2:** Descriptive Statistics (Adolescents from Add Health Wave II/1996).

Variables	Mean	Std. Dev.	Min	Max	N
*Parental Characteristics*					
Mom College (1994)	0.269	0.447	0	1	3898
Dad College (1994)	0.231	0.422	0	1	3898
Log of Income (1994)	3.634	0.693	0	6.907	3898
Plays Sports (1994)	0.147	0.354	0	1	3898
Allow to decide TV Time	0.839	0.367	0	1	3898
Set Bed Time	0.974	0.332	0	1	3898
Allow to decide what to eat	0.843	0.364	0	1	3898
Moved because of school district (1994)	0.412	0.492	0	1	3898
Child age when moved (1994)	8.016	5.643	0	17	3898
*Demographics*					
Age	16.261	1.557	12	18	3898
Male	0.487	0.500	0	1	3898
Grade	10.332	1.391	7	12	3898
White	0.653	0.476	0	1	3898
Black	0.189	0.392	0	1	3898
Hispanic	0.146	0.354	0	1	3898
Asian	0.096	0.294	0	1	3898
Other	0.013	0.113	0	1	3898
Lives with both biological parents	0.575	0.494	0	1	3898
School weight Problem	0.507	0.500	0	1	3898

### Measures

#### Dependent Variables

We consider eight weight-related behaviors: (i) Exercise – a binary variable indicating whether the respondent reported exercising three or more times during the past seven days; (ii) Sports – a binary variable indicating whether the individual reported playing an active sport such as baseball, softball, soccer, swimming or football; (iii) Hours of television/video viewing - the weekly hours of television viewing, including watching videos and playing video games; (iv) Sleep six hours or less – a binary variable indicating whether the individual usually gets six or fewer hours of nightly sleep; (v) Breakfast – a binary variable indicating whether the individual usually eats breakfast on weekday mornings; (vi) Fast food – the number of days in the past week the adolescent ate in a fast food restaurant such as McDonald's, Taco Bell, or KFC; (vii) Five Servings of Fruits or Vegetables – a binary variable indicating whether the adolescent had at least five servings of fruits or vegetables the previous day; (viii) Calorie-dense Snack – a binary variable indicating whether the adolescent consumed calorie-dense snacks (doughnuts, sweet rolls, muffins, pastries, cookies, brownies/pie, etc.) the previous day.

#### Independent Variables

The members of a social network are identified based on the set of close friends nominated by the respondent. For each adolescent we constructed measures of the percentages of his or her friends who exercise, play sports, get six or fewer hours of sleep, and regularly have breakfast, and measures of their average reported hours of television viewing and average number of days of eating in a fast food restaurant. We examine the robustness of the link between peer and individual behaviors to various controls, including demographic characteristics of the individual (age, gender, education and race) and whether the adolescent had been taught about the problems related to being overweight or underweight in school. In addition, using the data from the parent survey, we employed controls for a number of parental characteristics, including whether the adolescent lives with both biological parents, the parents' level of education, whether both parents work full time, family income, whether the parents chose their residence because of the school district and how old the adolescent was when they first moved into the district. Other parental measures considered are whether the parent plays an active sport (in the exercise and sport models), whether the parent allows the adolescent to decide how much television to watch (in the hours of television model), whether the parent allows the adolescent to decide when to go to bed (in the sleep model), and whether the parent allows the adolescent to decide what to eat (in the eating behaviors models). Hereafter, we refer to the latter indicators as parental weight-related activities. Finally, we use information on individuals' current and past BMIs, along with the current and past average BMIs of the peer group. We also estimated our models with BMI percentiles and BMI z-scores and the results were consistent with the ones reported here. We report the results with BMI to be consistent with the previous literature and to make our results comparable to them [18].

### Ethics Statement

We are registered and approved users of the Add Health dataset. As a part of the process for acquiring the data we underwent IRB review and received approval from the Institutional Review Board of the University of Toledo (2007). We are in no way using human or animal subjects directly (we are analyzing pre-existing data), thus written consent was not necessary. We have successfully completed our training on human subjects research review as well as HIPAA.

### Statistical Analyses

We estimate linear regression and probit models for adolescents' weight-related behaviors. In our most comprehensive model, the propensity to participate in weight-related behaviors by individual *i* in school *s* during time *t*, is given by
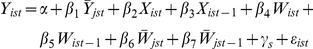
where 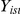 and 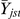 refer to the individual's participation in weight-related activities and peer group outcomes, respectively, measured in 1996. The vectors of individual and family characteristics measured in 1994 and 1996 are denoted by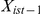 and 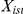; the variables 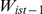 and 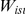 are the individual's own BMI in 1994 and 1996; 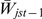 and 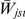 are the 1994 and 1996 peer group BMI; and 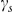 is a vector of school dummies. We measure the role of peer influence on the individual's weight-related behaviors, *β_1_* by estimating relatively parsimonious models and then consider models with an increasingly large set of controls to assess the robustness of the estimated peer effects.

Identifying social network effects in observational data is not without its challenges. First, there is the concern about confounding resulting from non-random selection of friends. Second, unobserved characteristics in the shared environment that affect all individuals in the social network may also cause environmental confounding. These confounding factors, if unaccounted for, can cause correlations suggestive of social network effects when none are present. We address this problem by estimating models that control for a large set of individual- and family-level factors, as well as school-level fixed effects, which capture unobserved characteristics common to adolescents from the same school and any other unmeasured school-specific influences. For example, schools will differ in the proximity to fast food restaurants, a well documented determinant of students' poor nutrition and overweight [33], as well as in the availability of vending machines, meal plans, and opportunities to expend energy (built environment, exercise facilities, etc.).

In addition to models with standard individual- and family-level controls (demographics, parents' education, etc.), we estimate models that control for individuals' and their peers' current (1996) and previous (1994) BMIs in order to control for the selection of friends based on weight status [18], [19], [34]. Peer BMI may proxy for the group's weight norm [17] and directly influence adolescents' exercise and food choices. We also utilize data on whether the family moved recently and whether the neighborhood was selected for the school district. Accounting for parental location preferences may further reduce the bias from non-random selection of friends [22], [27].

As mentioned earlier, there is no consensus in the literature as to the most appropriate methodology to account for non-random peer selection. Some studies have argued for using a measure of social network at a more exogenous level, such as the neighborhood or school level [27]. However, a problem with this approach is that social networks at such aggregated levels might not be relevant, i.e., these might not be the networks that influence individual behaviors [27]. Another suggested approach is to account for parental location preferences along with school-level fixed effects and controls for certain outcomes that might be driving the selection [18], [27]. For example, in the obesity and peer effects literature [18], individuals and their peers' lagged and contemporaneous body weights have been used to account for peer selection that might be driven by body weights. In another literature that focuses on risky health behaviors among adolescents such as drinking and drug use [27], peer selection was accounted for by controlling for parental location preferences and school-level fixed effects. The rationale is that for adolescents it is primarily the parents' location decision that determines with whom their children associate. To the extent that this is true, unobserved environmental confounders and factors that might drive residential location preferences can account for peer selection.

Our empirical strategy is a combination of these approaches. Specifically, we include individual and their peers' BMI to account for peer selection based on body weight, while acknowledging that body weight is not the only factor driving friendship selection. In addition, our controls for school-level fixed effects and parental location preferences attempt to account for that possibility that parents' location decisions affect peer selection.

## Results

The estimates of the peer effects from various models without school-level fixed effects are shown in Table 3, and the estimates with school-level fixed effects are reported in Table 4. For the binary outcomes, we report marginal effects estimated from probit models at the mean values of the independent variables. The hours of television viewing and the number of days eating in a fast food restaurant are treated as continuous variables, and the corresponding estimates are based on linear regression models.

**Table 3:** Estimated Friendship Network Effects (without school–level Fixed Effects).

	MODELS						
BEHAVIORS	(1)	(2)	(3)	(4)	(5)	(6)	(7)
Exercise	0.083**(0.019)	0.081**(0.018)	0.081**(0.018)	0.080**(0.019)	0.094**(0.024)	0.094**(0.024)	0.094**(0.024)
Sports	0.208**(0.020)	0.205**(0.019)	0.204**(0.020)	0.204**(0.020)	0.212**(0.024)	0.211**(0.024)	0.209***(0.024)
Hours of TV	0.072**(0.022)	0.062**(0.022)	0.060**(0.022)	0.059**(0.022)	0.102**(0.030)	0.100**(0.029)	0.100**(0.029)
Sleep six or fewer hours	0.022(0.014)	0.022(0.014)	0.019(0.013)	0.018(0.013)	0.025(0.016)	0.022(0.016)	0.022(0.016)
Breakfast	0.083**(0.017)	0.079**(0.017)	0.079**(0.017)	0.073**(0.017)	0.076**(0.021)	0.074**(0.021)	0.074**(0.021)
Fast Food	0.189**(0.019)	0.186**(0.019)	0.188**(0.020)	0.189**(0.019)	0.235**(0.024)	0.235**(0.024)	0.234***(0.024)
Five Servings of Fruits or Vegetables	0.080**(0.019)	0.074**(0.019)	0.073**(0.019)	0.072**(0.019)	0.067**(0.023)	0.067**(0.023)	0.067**(0.023)
Calorie-Dense Snacks	0.049**(0.019)	0.049**(0.019)	0.048**(0.019)	0.049**(0.019)	0.069**(0.023)	0.065**(0.024)	0.066**(0.024)
N	3898	3898	3898	3898	2760	2760	2760

*Notes:*

**sig at 1%.

**Table 4:** Estimated Friendship Network Effects (with school–level Fixed Effects).

	SPECIFICATION						
BEHAVIORS	(1)	(2)	(3)	(4)	(5)	(6)	(7)
Exercise	0.064**(0.019)	0.062**(0.019)	0.062**(0.020)	0.062**(0.020)	0.080**(0.025)	0.080**(0.025)	0.079**(0.025)
Sports	0.185**(0.021)	0.183**(0.021)	0.181**(0.021)	0.181**(0.021)	0.187**(0.025)	0.187**(0.025)	0.184***(0.025)
Hours of TV	0.014(0.023)	0.011(0.023)	0.009(0.023)	0.009(0.023)	0.051(0.030)	0.047(0.030)	0.048(0.030)
Sleep six or fewer hours	−0.001(0.002)	−0.001(0.002)	−0.001(0.002)	−0.001(0.002)	0.001(0.001)	0.001(0.001)	0.001(0.001)
Breakfast	0.022*(0.010)	0.021*(0.011)	0.021*(0.10)	0.018†(0.010)	0.014(0.010)	0.012(0.011)	0.013(0.011)
Fast Food	0.131**(0.020)	0.131**(0.020)	0.131**(0.020)	0.132**(0.020)	0.178**(0.025)	0.178**(0.025)	0.178***(0.025)
Five Servings of Fruits or Vegetables	0.032†(0.019)	0.028(0.019)	0.028(0.019)	0.028(0.019)	0.027(0.023)	0.027(0.023)	0.027(0.023)
Calorie- Dense Snacks	0.020(0.019)	0.019(0.020)	0.020(0.019)	0.022(0.019)	0.027(0.025)	0.023(0.025)	0.021(0.025)
N	3898	3898	3898	3898	2760	2760	2760

*Notes:*

**sig at 1%;

*sig at 5%;

†sig at 10%.

Column 1 of Table 3 presents estimates from our baseline model, which controls only for the adolescents' demographics. Comparison of the results from the baseline model to models that control for individual- and family-level factors allows us to investigate the robustness of the estimated network effects. In particular, the model in column 2 also controls for parental characteristics (including parental education and income and whether the individual lives with both biological parents). In column 3, a measure for whether the adolescent was taught about the problems related to being overweight or underweight in school is added, together with other parental measures, including whether the current residential location was chosen because of the school district, how old the respondent was when he/she moved to the current location, and indicators for parental activities. The model in column 4 adds the individuals' own lagged BMIs, and models in columns 5–7 also include peers' lagged BMIs, peers' contemporaneous BMIs and individuals' own contemporaneous BMIs. We report only the estimated peer effects in Tables 3 and 4, and we report the full sets of estimates based on the most comprehensive models (column 7) in Tables 5 and 6. (The estimates from models without school-level fixed effects are in Table 5, and the estimates from models with school-level fixed effects are in Table 6.)

**Table 5:** Detailed Estimates from Full Specification (without school–level Fixed Effects).

	Exercise	Sports	Hours of TV	Sleep	Breakfast	Fast Food	Fruits or Vegetables	Calorie-Dense Snack
**Variables**								
BMI	−0.004(0.005)	−0.010*(0.005)	0.128(0.142)	−0.001(0.002)	0.005(0.003)	−0.012(0.015)	0.001(0.005)	−0.007(0.005)
BMI (1994)	0.007(0.006)	0.012*(0.005)	0.112(0.161)	0.002(0.002)	−0.005(0.003)	−0.021(0.018)	−0.002(0.006)	−0.009(0.006)
*Peer Measures*								
Peer Activity	**0.094** **(0.024)	0.209***(0.024)	**0.100** **(0.029)	**0.022**(0.016)	**0.074** **(0.021)	0.0234***(0.024)	**0.067** **(0.023)	**0.066** **(0.024)
Peer BMI	−0.001(0.004)	−0.001(0.005)	−0.009(0.087)	0.001(0.001)	−0.002(0.002)	0.014(0.013)	−0.013**(0.004)	−0.007*(0.004)
Peer BMI (1994)	0.004(0.004)	0.001(0.004)	0.010(0.121)	0.001(0.001)	−0.002(0.003)	−0.004(0.015)	0.004(0.004)	0.003(0.004)
*Parental Characteristics*								
Mom College (1994)	0.023(0.029)	0.005(0.030)	−1.129†(0.051)	0.001(0.011)	−0.011(0.018)	0.188*(0.091)	0.028(0.028)	0.035(0.029)
Dad College (1994)	0.078*(0.031)	0.083*(0.033)	−1.415*(0.068)	−0.002(0.012)	0.041*(0.018)	−0.092(0.098)	0.016(0.031)	0.014(0.032)
Log of Income (1994)	−0.023(0.018)	−0.013†(0.018)	−0.874†(0.475)	−0.002(0.006)	0.021†(0.011)	−0.077(0.058)	0.001(0.017)	−0.004(0.018)
Plays Sports (1994)	0.014(0.030)	0.058†(0.032)	-	-	-	-	-	-
Allow to decide TV Time	-	-	1.296(0.841)	-	-	-	-	-
Set Bed Time	-	-	-	−0.017(0.044)	-	-	-	-
Allow to decide what to eat	-	-	-	-	−0.033(0.018)	0.031(0.018)	−0.027(0.032)	0.027(0.034)
Moved because of school district	0.048*(0.023)	−0.013(0.024)	−0.395(0.554)	0.015†(0.009)	−0.001(0.014)	−0.002(0.014)	0.054*(0.022)	0.002(0.023)
Child age when moved	0.004†(0.002)	−0.002(0.002)	−0.037(0.055)	0.001(0.001)	−0.002(0.001)	0.015*(0.007)	0.001(0.002)	0.004†(0.002)
*Demographics*								
Age	−0.028(0.018)	−0.026(0.019)	−0.551(0.465)	0.016*(0.007)	−0.013(0.011)	0.159**(0.060)	0.002(0.017)	0.008(0.019)
Male	0.013(0.023)	0.222**(0.024)	3.236**(0.564)	−0.15†(0.006)	0.014(0.014)	0.167*(0.074)	0.022(0.022)	0.112**(0.023)
Grade	−0.011(0.020)	−0.047†(0.020)	−0.438(0.488)	−0.005(0.008)	0.005(0.012)	0.043(0.065)	−0.024(0.018)	−0.021(0.020)
White	−0.016(0.053)	0.142**(0.053)	−1.045(1.291)	−0.001(0.020)	0.015(0.033)	−0.046(0.157)	−0.104*(0.052)	−0.032(0.053)
Black	−0.051(0.061)	0.072(0.062)	4.782**(1.532)	0.023(0.029)	−0.023(0.040)	0.312†(0.181)	0.007(0.026)	0.065(0.060)
Lives with both biological parents	0.011(0.027)	0.009(0.028)	0.232(0.661)	0.001(0.009)	0.004(0.017)	−0.009(0.085)	0.007(0.026)	0.033(0.060)
School Weight Program	0.003(0.024)	0.025(0.025)	−1.236*(0.567)	−0.009(0.008)	0.020(0.014)	0.089(0.076)	0.031(0.023)	−0.001(0.024)

*Notes:*

**sig at 1%;

*sig at 5%;

†sig at 10%.

**Table 6:** Detailed Estimates from Full Specification (with school–level Fixed Effects).

	Exercise	Sports	Hours of TV	Sleep	Breakfast	Fast Food	Fruits or Vegetables	Calorie-Dense Snacks
**Variables**								
BMI	−0.003(0.006)	−0.008(0.006)	0.139(0.144)	−0.001(0.002)	0.001(0.004)	−0.009(0.016)	0.001(0.005)	−0.004(0.006)
BMI (1994)	0.007(0.006)	0.009(0.006)	0.082(0.167)	0.001(0.003)	−0.006(0.004)	−0.026(0.019)	−0.001(0.006)	−0.012†(0.006)
*Peer Measures*								
Peer Activity	**0.079** **(0.025)	0.184***(0.025)	**0.048**(0.030)	**0.001**(0.001)	**0.013**(0.011)	0.178***(0.025)	**0.027**(0.023)	**0.021**(0.025)
Peer BMI	−0.002(0.004)	−0.003(0.004)	−0.021(0.092)	0.001(0.002)	−0.003(0.002)	0.005(0.013)	−0.014**(0.004)	−0.008*(0.004)
Peer BMI (1994)	0.008(0.005)	0.002(0.005)	0.113(0.131)	0.002(0.002)	−0.001(0.003)	−0.006(0.015)	0.007(0.005)	0.002(0.005)
*Parental Characteristics*								
Mom College (1994)	0.029(0.031)	0.013(0.033)	−0.795(0.696)	−0.002(0.015)	−0.006(0.021)	0.145(0.095)	0.033(0.030)	0.030(0.031)
Dad College (1994)	0.084*(0.033)	0.078*(0.034)	−1.246†(0.739)	0.012(0.019)	0.036†(0.021)	−0.080(0.105)	0.016(0.033)	0.012(0.035)
Log of Income (1994)	−0.032(0.020)	−0.027(0.021)	−0.289(0.549)	−0.003(0.009)	0.019(0.014)	−0.082(0.065)	−0.041*(0.019)	0.004(0.020)
Plays Sports (1994)	0.009(0.032)	0.057†(0.034)	-	-	-	-	-	-
Allow to decide TV Time	-	-	1.322(0.879)	-	-	-	-	-
Set Bed Time	-	-	-	−0.027†(0.020)	-	-	-	-
Allow to decide what to eat	-	-	-	-	−0.036(0.021)	0.032(0.021)	−0.036(0.021)	0.019(0.036)
Moved because of school district	0.029(0.026)	−0.022(0.002)	−0.147(0.628)	0.022*(0.012)	0.013(0.017)	−0.028(0.083)	0.013(0.017)	0.010(0.026)
Child age when moved	0.003(0.002)	−0.002(0.002)	−0.048(0.058)	0.002†(0.001)	−0.001(0.002)	0.010(0.007)	−0.001(0.002)	0.004(0.002)
*Demographics*								
Age	−0.014(0.020)	−0.021(0.020)	−0.570(0.481)	0.022*(0.009)	−0.013(0.013)	0.145*(0.062)	0.016(0.019)	0.012(0.020)
Male	0.018(0.025)	0.234**(0.026)	3.240**(0.597)	−0.025*(0.012)	0.020(0.017)	0.165*(0.078)	0.041†(0.024)	0.097**(0.025)
Grade	−0.029(0.022)	−0.028(0.023)	−0.063(0.513)	−0.005(0.011)	0.003(0.015)	−0.027(0.021)	−0.027(0.021)	−0.018(0.023)
White	−0.019(0.056)	0.078(0.060)	−0.604(0.400)	0.004(0.025)	0.020(0.041)	−0.107†(0.059)	−0.107†(0.059)	−0.036(0.058)
Black	−0.057(0.069)	0.094(0.073)	2.910*(1.703)	0.062†(0.047)	0.009(0.044)	−0.146*(0.056)	−0.146*(0.056)	0.104(0.067)
Lives with both biological parents	0.010(0.029)	0.009(0.030)	−0.049(0.703)	−0.002(0.013)	0.010(0.019)	0.027(0.028)	0.027(0.028)	0.027(0.029)
School Weight Program	0.005(0.027)	0.030(0.028)	−1.150(0.650)	−0.017(0.013)	0.085(0.083)	0.034(0.026)	0.034(0.026)	−0.004(0.027)

*Notes:*

** sig at 1%;

* sig at 5%;

† sig at 10%.

The estimates from the baseline model, which includes only peer and demographic variables (see column 1 of Table 3), suggest that having friends who are more engaged in weight-related behaviors is associated with an increase in individuals' participation in these activities. This result holds for all weight-related behaviors except for sleeping six or fewer hours. The effects are sizeable across behaviors, with peer influence having the greatest effect on participation in a sport and eating at fast food restaurants. After family-level information is added, the estimates in columns 2 and 3 decrease in magnitude slightly. The greatest change in the magnitudes occurs in model 5, where we add peers' lagged BMIs to account for the selection of friends. The estimated coefficients change little after peers' contemporaneous BMIs and individuals' own contemporaneous BMIs are included in columns 6 and 7, respectively. In the most comprehensive model in Table 3 (column 7), we find positive and statistically significant associations for all behaviors except for getting six or fewer hours of sleep.

The fact that the estimates of the peer influence change in magnitude across different model specifications is consistent with the idea that there are confounding factors that can bias the effect of peers on individuals' behaviors. However, we cannot rule out that even the most comprehensive model in Table 3, model 7, overstates the peer effects because of other confounding factors for which we do not account. In an attempt to minimize the bias further, we re-estimate all models in Table 3 with school-level fixed effects. The corresponding estimates in Table 4 suggest that only exercise, sports, and eating at fast food restaurants have consistently positive and significant social network effects after accounting for unmeasured school-specific influences. The estimated effects in Table 4 are smaller than those in Table 3, suggesting that the unmeasured heterogeneity across schools introduces upward bias in the peer effects estimates.

The results from our most comprehensive model, Model 7, in Table 4 suggest that, on average, a 10 percentage points increase in the proportion of friends who exercise is associated with a 0.79 percentage points (p = 0.025) greater likelihood that the individual exercises. The effect of a same-size increase in the proportion of friends who participate in an active sport is a 1.84 percent points (p<0.001) greater likelihood that the individual participates in an active sport. A one-day increase in the average number of weekdays friends eat at fast food restaurants is associated with a 0.18 (p<0.001) increase in the number of days the adolescent eats in a fast food restaurant. The estimated peer effects associated with these three activities change little across specifications, as shown in Table 4. We find no evidence that hours of TV viewing, sleeping six or fewer hours, eating breakfast, eating five servings of fruits/vegetables or consuming calorie-dense snacks is affected by friends' engagement in such activities when unobserved school-level factors are accounted for. However, the coefficients on all these activities are positive and it is possible that we cannot reject the null hypothesis of no effect due to the sampling error. It is also important to note that the weight-related behaviors were not significantly associated with the BMI (Table 6); this could potentially be due to unobserved heterogeneity (e.g. genetic predisposition) and from having BMI in 1994 and 1996 highly correlated.

As a further robustness check of our estimates we performed a sensitivity analysis by restricting the sample to only those with stable friendship links between waves 1 and 2, i.e. the individual who were nominated as friends in both the waves. The rationale for this analysis was to test whether our estimates (after accounting for peer selection) were driven by a change in friends' pool composition between the waves. These restrictions lead to a reduction of the sample size by 199 from our preferred specification. However, it is important to note here that Add Health did not allow us to indentify whether this reduction in the sample was because these individuals were no longer friends or whether the nominated friends were no longer part of the survey. The estimates from the restricted sample were quantitatively very similar to the estimates presented above, suggesting that our results are not driven by a change in the friendship network composition.

## Discussion

This study investigates the spread of weight-related behaviors in adolescents' friendship networks. Utilizing a longitudinal research design and a representative sample from the Add Health, we examine the influence of peer behaviors on adolescents' engagement in eight weight-related behaviors. We find significant peer effects for pursuing an active sport, regular exercise, and the frequency of eating in fast food restaurants, suggesting that an individual is more likely to engage in these behaviors if his or her friends do.

We find no consistent evidence in support of peer effects on TV viewing, sleeping six or less hours, or on eating breakfast, calorie-dense snacks, or five servings of fruits and vegetables. Adolescents engage in these activities primarily in their parents' homes, which limits both the extent to which these behaviors are self-directed by the adolescent as well as how observable they are to friends; this is especially true for hours of sleep and breakfast consumption. In addition, we find that the association between friends' average TV viewing and that of the individual declines considerably and becomes statistically insignificant after accounting for unobserved factors at the school level. A similar result is obtained for unhealthy snack food consumption, another activity where peer influences are plausible. Our findings for TV viewing complement Fletcher [32], who documented a sizable association between school-level and individual-level TV viewing. We find weaker and statistically insignificant associations after accounting for school-level unobserved heterogeneity. While the estimates are not directly comparable across studies since we use close friends as reference group rather than all schoolmates, our results cast doubt on the presence of peer effects on TV viewing.

Our study contributes to the emerging literature on the potential mechanisms by which obesity may spread within social networks [17]–[22]. It also has significant implications for understanding the influence of person-specific behaviors that are related to obesity in adolescence, a time when individuals become vulnerable to weight gain and peer influence. Determining the weight-related behaviors most salient to peer influence has direct policy implications by suggesting the behaviors within social networks that should be the focus of interventions.

Our findings indicate that any policy intervention that alters the weight-related behavior of an individual who is embedded in a social network might also have an indirect effect on the behavior of untreated adolescents who are in the same social network [35]. It may be fruitful to complement traditional weight reduction interventions with strategies that focus on harnessing peer support to modify behaviors and, especially among adolescents, strategies that target norms regarding sports, exercise, and eating fast food. Given that these behavioral norms will tend to differ across groups, the effectiveness of an intervention will likely be enhanced by taking into account the particular peer environment. By reducing calories from fast food and increasing physical activity levels among adolescents, such interventions may help reverse current trends in adolescent obesity. Finally, the findings of our study are consistent with the idea that changing physical activity and food norms has contributed to the spread of obesity. However, this consistency does not rule out weight norms as additional channel [17], [18]. Also, to the extent that these results reflect peer selection, (i.e. the observed associations might themselves be a product of peer selection) they might be of limited use as policy levers aimed at encouraging healthy body weights.

Several limitations of our research warrant elaboration. Even after accounting for common unobserved influences at the school level, the relationships that we found to be significant may be driven in part by the correlated effects within smaller groups. This is a concern since the individual selects his or her friends, who will tend to be similar to the individual. Although we utilize both lagged and contemporaneous measure of body weight along with school-level fixed effects and parental location preferences, it is likely that selection could be conditioned on other unobserved characteristics, such as degree of risk aversion. If common attributes and environmental factors that influence adolescents' weight-related behaviors are not captured by our individual- and family-level controls or by the school-level fixed effects, the estimates presented here may overstate the influence of friend networks. In that regard, future studies should investigate further how friendship ties are formed. For example, future research could examine whether friendship ties are more likely to form between individuals with similar observable traits after controlling for demographics and environmental confounders [36]. On a similar note it might also be worthwhile to examine whether the spread of one behavior in social networks (e.g. fast-food consumption) might influence the spread of another (e.g. unhealthy snacking). This would be similar to the Mednick et al. [37] study which found that the spread of sleep patterns in social networks also have an impact on marijuana consumption in the social networks. In addition, future study should look into whether peer effects in obesity operate via pathways other than the ones examined here, for example, economic insecurity [38]. Another potential limitation of the current study is our reliance on self-reported behaviors. However, to the extent that the measurement error in the peer group activities is classical, the estimated magnitudes of the peer effects will be conservative.
